# Synthesis, Properties, and Electrochemical Performance of ϕ_y_‑type KCu_2_OH(MoO_4_)_2_(H_2_O)

**DOI:** 10.1021/acs.inorgchem.6c01466

**Published:** 2026-09-01

**Authors:** Walajhone Oliveira Pereira, Déborah E. de F. Sodré, Márcio A. P. Almeida, Auro A. Tanaka, Clenilton C. dos Santos, Rossano Lang, Alan S. de Menezes, Adenilson Oliveira Dos Santos

**Affiliations:** † Center for Social Sciences, Health, and Technology, 37892 Federal University of Maranhão, Imperatriz, Maranhao 65900-410, Brazil; ‡ Physics Department, Federal University of Maranhão, CCET, São Luís, Maranhão 65080-805, Brazil; § Postgraduate Program in Aerospace Engineering, Federal University of Maranhão, São Luís, Maranhão 65080-805, Brazil; ∥ Department of Chemistry, 37892 Federal University of Maranhão, São Luís, Maranhão 65080-805, Brazil; ⊥ Institute of Science and Technology, 28105 Federal University of São Paulo (UNIFESP), São José dos Campos, São Paulo 12231-280, Brazil

## Abstract

A new KCu_2_OH­(MoO_4_)_2_(H_2_O) compound was synthesized via the sonochemical method. Its crystal structure was identified by powder X-ray diffraction (PXRD) and Rietveld refinement, revealing a polymorph of layered transition-metal molybdates (ϕ_*y*_). The compound crystallizes in a trigonal system with *R*3̅ space group with lattice parameters: *a* = *b* = 6.0320(3) Å, *c* = 21.3445(6) Å and α = β = 90°, γ = 120°. Morphology, compositional, textural, and thermal properties were evaluated by scanning electron microscopy (SEM), energy-dispersive X-ray spectroscopy (EDS), N_2_ physisorption, and thermogravimetric analysis (TGA). Vibrational properties were investigated by FT-IR and Raman spectroscopies. Additionally, cyclic voltammetry, galvanostatic charge–discharge, and electrochemical impedance spectroscopy (EIS) were used to characterize its electrochemical behavior. The material exhibits a specific capacitance of 967 F/g at a current density of 1 A/g. Continuous charge/discharge studies demonstrated a moderate cycling stability over the first 1,000 cycles. The findings suggest a mixed charge-storage mechanism involving both diffusion-controlled Faradaic reactions and pseudocapacitive contributions. Further studies are required to validate KCMO as a viable material for electrochemical energy-storage devices.

## Graphical abstract

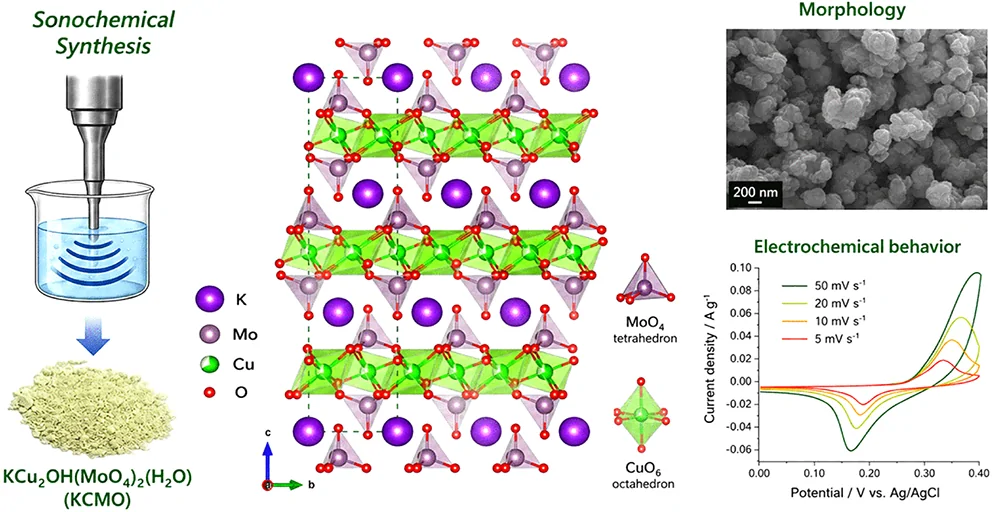

## 1 Introduction

Layered hydroxides are a special class of material in which the crystalline structures are built through the stacking of two-dimensional electrically charged or neutral units, which are connected by electrostatic forces and hydrogen bonds, and are particularly suitable for intercalation reactions and surface functionalization.^1,2^ The importance of layered compounds lies in their ability to retain chemical species with compatible electric charges, allowing expansion or contraction of the dimension along the basal axis, usually perpendicular to the layers. At the same time, the overall structure is maintained.^3^

In recent years, transition metal molybdates have garnered considerable interest in various scientific and technological fields, owing to their unique photoluminescent,^4,5^ photocatalytic,^6,7^ magnetic^8,9^ functionalities, as well as their promising performance in electrochemical energy storage. They have been applied as anodes for lithium batteries and supercapacitors,^10,11^ exhibit antibacterial activity,^12,13^ and are also used as corrosion inhibitors for iron and its alloys,^2^ among other uses. Therefore, the synthesis of layered transition metal molybdates (LTMs) has been of great interest due to their wide range of properties and intercalation chemistry, potentially accessible internal surface area, and use as precursors for the generation of 2D nanosheets.^14,15^

LTMs have the general formula AT_2_OH­(MoO_4_)_2_(H_2_O), where A = NH_4_^+^, Na^+^ or K^+^ and T = Mn^2+^, Fe^2+^, Co^2+^, Ni^2+^, Cu^2+^, Zn^2+^ or combinations of these metals. Two structural polytypes, designated as ϕ_*x*_ and ϕ_*y*_, were first proposed by Pezerat^16^ in the 1960s. Later, Clearfield *et al*.^17^ (ϕ_*x*_) in 1967 and Levin *et al*.^18^ (ϕ_*y*_) in 1996 solved their structures. In both phases, the divalent metal occupies octahedral sites, while molybdate groups are tetrahedrally coordinated, forming stacked sheets held together by van der Waals interactions.^15^

The ϕ_*x*_ phase is characterized by octahedra chains of transition metals connected by tetrahedral molybdate groups, while in the ϕ_*y*_ phase, the octahedra of transition metals share edges forming layers.^19,20^ Furthermore, the ϕ_*x*_ phase is typically monoclinic, with space group *C*2/*m*, while the ϕ_*y*_ phase crystallizes in a trigonal system, with *R*3̅ space group.^21^ (Table S1 Supporting Information) summarizes LTMs from known series in the literature. Besides, trimetallic compounds such as (NH_4_)­H_2_Co_2_O­(OH)­(MoO_4_)_1.6_(WO_4_)_0.4_·H_2_O^20^ and (NH_4_)­Ni_2.4_Co_0.6_O­(OH)­(MoO_4_)_2_·1.5H_2_O^22^ were also investigated, describing the ϕ_*y*_ phase. On the other hand, LTMs with ϕ_*x*_ structures have also been reported in synthesis with the monovalent cations NH_4_^+^, Na^+^, and K^+^; however, ϕy was observed only in ammonium-containing systems.^14,23,24^

In particular, transition metal molybdates are known as potential electrodes in energy storage devices due to their wide range of oxidation states (from Mo^6+^ to Mo^0^), which results in a high theoretical capacity, as well as excellent electrochemical activity, good electrical conductivity, low cost, easy synthesis, good availability, and eco-friendly nature.^25,26^ In this context, several recent studies have focused on developing high-performing supercapacitor electrodes to address the increasing demand for sustainable energy sources.^27–32^

As far as we know, LTMs containing monovalent cations other than ammonium have only been reported in the monoclinic ϕ_*x*_ phase. This work reports a novel molybdate, KCu_2_OH­(MoO_4_)_2_(H_2_O), which represents the first potassium-based LTM belonging to the trigonal ϕ_*y*_ structural family. Comprehensive characterizations, including powder X-ray diffraction (PXRD), scanning electron microscopy (SEM), energy-dispersive X-ray spectroscopy (EDS), N_2_ physisorption, and thermogravimetric analysis (TGA), were performed to evaluate the crystal structure, morphology, elemental composition, textural features, and thermal stability. Vibrational properties were investigated using infrared and Raman spectroscopies to assess chemical bonding. Additionally, electrochemical analyses were conducted to evaluate the charge-storage behavior and potential energy-storage applications.

## 2 Materials and Methods

### 2.1 Sample Synthesis

The material was synthesized via a sonochemical method. Initially, two separate aqueous solutions were prepared: 2 mmol of K_2_MoO_4_ (Sigma-Aldrich, 99%) dissolved in 10 mL of distilled water, and 1 mmol of CuCl_2_·2H_2_O (Sigma-Aldrich, 99%) dissolved in 10 mL of distilled water. A 19 kHz ultrasonic probe was immersed in the CuCl_2_·2H_2_O solution, and the K_2_MoO_4_ solution was added dropwise while the probe was continuously sonicated. The mixture was sonicated for 30 min, yielding a greenish colloidal suspension. The suspension was then washed with distilled water and centrifuged at 5000 rpm (3040 g) several times to remove impurities and dried at 50 °C for 24 h in a hot-air oven. A photograph of the pale green powder obtained by this method is shown in (Figure S1 Supporting Information).

### 2.2 Characterization Techniques

PXRD measurement at room temperature was performed in a Bruker diffractometer, model D8 Advance, operating with Cu–Kα radiation (λ = 1.5418 Å), 40 kV/40 mA, Bragg-Bretano parafocal geometry, and LynxEye XE linear detector. The diffraction pattern was collected over the angular range 2θ from 8° to 100°, with an angular step of 0.02° and an acquisition time of 1 s. The PXRD pattern was refined by the Rietveld method using the GSAS II software^33^ with the crystallographic data [04–018–0438] from the Inorganic Crystal Structure Database (ICSD).

The morphology and elemental composition were investigated using a Zeiss scanning electron microscope (model EVO 15) equipped with an SE detector and a Bruker EDS detector (model XFlash 410-M). For that, the sample was directly conditioned on a conducting carbon double-sided tape and coated with an Au film.

Nitrogen physisorption (adsorption/desorption isotherms at 77 K) was obtained using a Quantachrome Instruments Nova 4200e. Before analysis, the samples were heat-treated at 130 °C for 4 h. The specific surface area (SBET) was estimated using the Brunauer–Emmett–Teller (BET) method. The pore size distribution was determined using the DFT method, considering a cylindrical pore NLDFT equilibrium model.

Fourier-transform infrared (FT-IR) spectrum was recorded on a KBr pellet using a Nicolet (model Nexus 470) spectrometer. Measurements were performed in the wavenumber range of 400–3900 cm^–1^, with 64 scans and a spectral resolution of ≈ 4 cm^–1^.

Raman spectroscopy measurements were carried out on a Horiba/Jobin-Yvon triple spectrometer, model T64000, equipped with a liquid-nitrogen-cooled CCD detector. A green solid-state laser (λ = 532 nm) with 50% filter intensity was focused on the sample surface with a 20× objective lens with a focal length of 20.5 mm. Spectra were acquired with 5 accumulations and a 60 s acquisition time. The slit widths were adjusted to obtain a spectral resolution of ≈ 2 cm^–1^.

Thermogravimetric analysis (TGA) was performed using a TA Instruments Q50 analyzer. A 5.62 mg sample was heated in an aluminum crucible from 303 to 1050 at 10 K/min under a nitrogen atmosphere at 100 mL/min.

The electrochemical measurements were made using an Autolab PGSTAT 302 N potentiostat (Metrohm Autolab), equipped with an FRA2 module and controlled by NOVA 2.1.3 Software. The equipment was used to characterize the supercapacitor behavior in a system with three electrodes in an aqueous solution of 3 M KOH, acting as the electrolyte. Pt wire and Ag/AgCl were used as counter and reference electrodes, respectively. The working electrode was prepared by depositing the synthesized material onto Ni foam, which served as the substrate. The mass ratio of the active material (KCMO), carbon black (Super P), and polyvinylidene fluoride (PVDF) was 8:1:1. The electrochemical characteristics were studied through cyclic voltammetry (CV) and galvanostatic charge/discharge measurements (QCD) at room temperature. From the analysis of QCD, the specific capacitances (*C*_s_) for each current density were calculated, according to eq 1:^34,35^

1 $$C_{s}=\frac{I\Delta t}{m\Delta V}$$

where *I* is the current (A), Δ*t* corresponds to the discharge time (s), *m* is the active material mass (mg), and ΔV is the potential window (V).

Electrochemical impedance spectroscopy (EIS) measurements were performed under the same experimental conditions as the CV and GCD measurements, using the active material deposited on Ni foam in 3 M KOH electrolyte. The data were collected at open-circuit potential (OCP) to evaluate the intrinsic impedance response of the electrode system under equilibrium conditions.

## 3 Results and Discussion

### 3.1 Structural Property

No matching diffraction pattern was found in the ICSD database for a hydrated molybdate compound containing potassium and copper. The closest match was identified using the PDF [04–018–0438] pattern,^23^ which describes the NH_4_H_3_Cu_2_Mo_2_O_10_ compound in the LTM class. Based on the qualitative analysis of the diffraction pattern, it was assumed that the obtained material is isostructural to the ϕ_*y*_ phase. Thus, its formula can be written as KCu_2_OH­(MoO_4_)_2_(H_2_O) (labeled as KCMO). Rietveld refinement was performed to test this hypothesis, in which all N atoms of the NH_4_ group were replaced by K. The experimental diffraction pattern refined by the Rietveld method is shown in Figure 1a.

**Figure 1:**
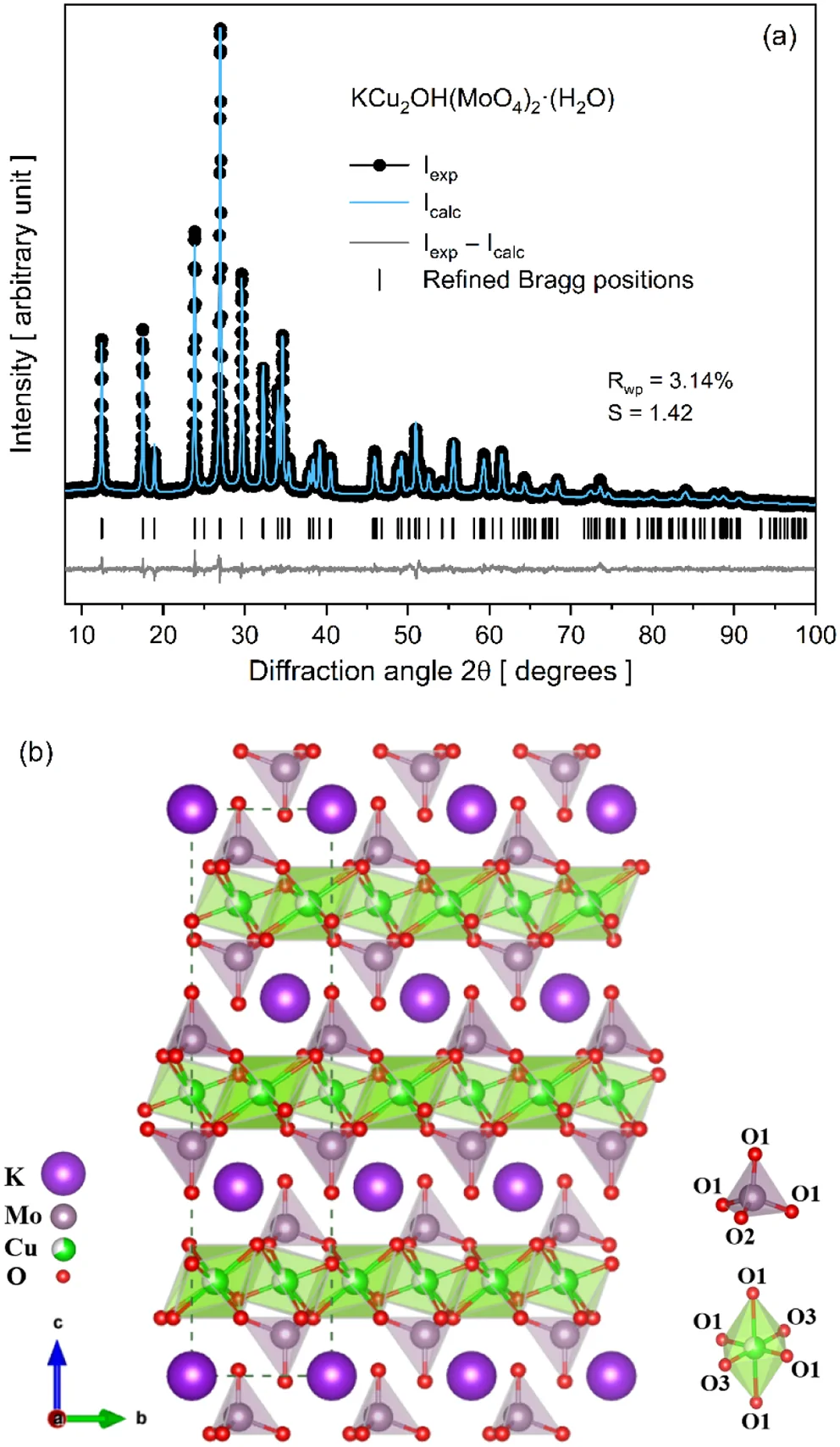
(**a**) PXRD pattern of KCMO refined by the Rietveld method. (**b**) Crystal structure of KCMO along the *a*-axis without hydrogen atoms.

The qualitative analysis and the *R*_wp_ = 3.14% and *S* = 1.42 accuracy indexes of the refinement attest that the obtained material is an LTM, with KCu_2_OH­(MoO_4_)_2_(H_2_O) formula and ϕ_*y*_ structure, which crystallizes in a trigonal system with $R3̅\left(C_{3i}^{2}\right)$ space group, containing three molecules per unit cell (Z = 3). Its lattice parameters are *a* = *b* = 6.0320(3) Å, *c* = 21.3445(6) Å, α = β = 90°, γ = 120° and V = 672.58(5) Å^3^.

The atomic positions, occupancy factors, and atomic displacement parameters obtained from the Rietveld refinement are given in Table 1. Additionally, relevant lengths and bond angles are shown in Table 2. Refinement results indicate that KCMO adopts a two-dimensional layered structure composed of edge-sharing {Cu^II^O_6_} octahedral units and {Mo^VI^O_4_} tetrahedra, as depicted in Figure 1b. Moreover, the refinement suggests that the occupancy factor of each Cu atom is 2/3, adopting a hexa-coordination with distorted octahedra with Cu–O distances in the range of 1.863(2)-2.436(5) Å.

**Table 1:** Atomic Positions, Occupancy Factors, and Atomic Displacement Obtained for KCMO with Rietveld Refinement

Atom	Wyckoff sites	X	Y	Z	S.O.F.	U_iso_ [Å^2^]
Mo1	6c	0	0	0.4061(1)	1	0.0584(6)
Cu1	9d	0.5	0	0.5	0.667	0.0234(8)
K1	3a	0	0	0	1	0.091(2)
O1	18f	0.133(1)	0.782(1)	0.1022(1)	1	0.052(2)
O2	6c	0	0	0.3237(5)	1	0.166(4)
O3	6c	0	0	0.4689(3)	1	0.016(2)

**Table 2:** Selected Lengths (Å) and Bond Angles (°) of the KCMO^a^

Bond	Bond length	Bond	Bond angle	Bond	Bond angle
Mo–O1	1.784(8)	O1–Mo–O2	110.6(3)	O1^iii^–Cu–O1^iv^	89.8(2)
Mo–O2	1.760(1)	O1–Mo–O1^i^	108.3(3)	Mo–O1–Cu	114.3(2)
Cu–O1	2.436(5)	O3^ii^–Cu–O3	179.9(1)	Mo–O1–K	116.8(2)
Cu–O3	1.863(2)	O3–Cu–O1^iii^	91.9(2)	Cu–O3–K	95.2(1)
K–O1	2.862(6)	O3–Cu–O1^iv^	101.6(2)	Cu–O3–Cu^i^	144.1(1)

a Symmetry transformations used to generate equivalent atoms: (i) – x+y, 1–x, z; (ii) 2/3–x, 1/3–y, 1/3–z; (iii) 1–y, 1+x–y, z; (iv) x, y–1, z.

According to Chuan-De Wu *et al.*,^23^ structural refinement indicates that approximately one-third of the copper sites are unoccupied, with the corresponding vacancies distributed randomly rather than in an ordered pattern. Nevertheless, a certain degree of ordering may exist within individual layers, while the stacking of these layers remains disordered.

### 3.2 Morphology and Elemental Composition

The SEM images presented in Figure 2a,b reveal that the material consists of aggregated particles with irregular to rounded morphologies and a moderate size variation. Consistent with the relatively broad diffraction peaks observed in the PXRD pattern, KCMO exhibits particles with sizes predominantly smaller than 200 nm. Particle-size analysis from SEM micrographs (Figure S2, Supporting Information) confirmed an average particle size of ≈ 163 ± 18 nm based on a Gaussian fit.

**Figure 2:**
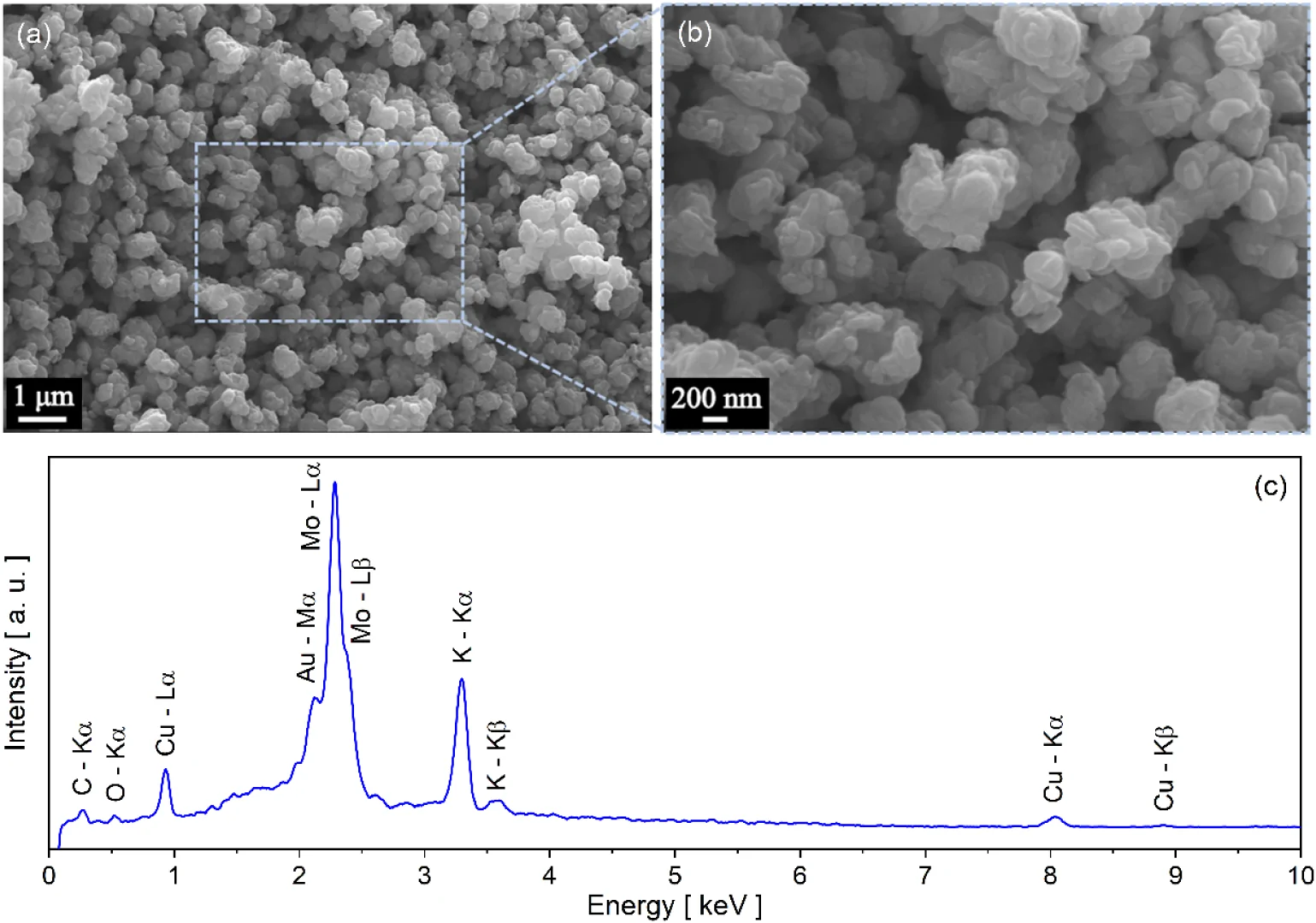
SEM images of KCMO at (**a**) low and (**b**) high magnification. (**c**) Corresponding EDS spectrum of the sample.

The EDS spectrum (Figure 2c) validates the elementary existence of Mo, K, Cu, and O atoms in the synthesized material. Signals corresponding to C (≈ 0.28 keV) and Au (≈ 2.12 keV) are also detected, originating from the tape used as a support for the measurements and metallic coating, respectively.

### 3.3 Textural Properties

Nitrogen adsorption/desorption measurements were carried out to investigate the textural properties of KCMO. The corresponding isotherms are shown in Figure 3. The material exhibits a relatively low specific surface area (SBET < 10 m^2^/g), implying a limited microporosity. The adsorption isotherm shows a hysteresis loop at high relative pressures, suggesting the presence of mesoporous domains mainly associated with irregular particle aggregation, in line with the SEM observations. Moreover, an increase in the adsorbed volume near p/p_0_ ≈ 1 suggests adsorption within larger mesoporous and interparticle voids.

**Figure 3:**
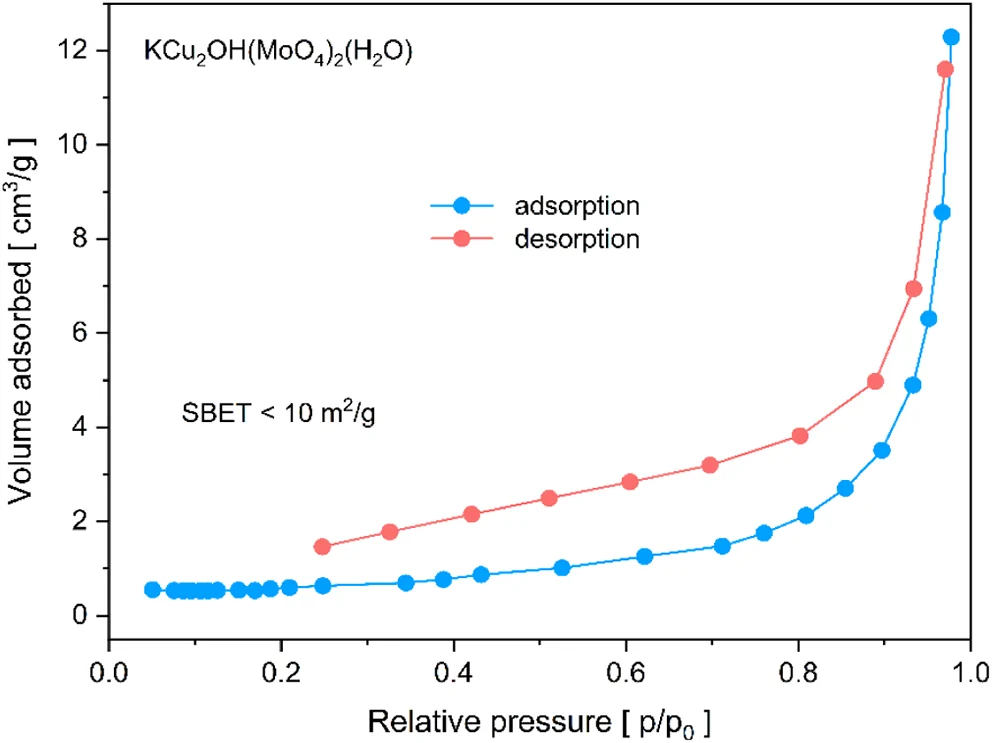
N_2_ adsorption/desorption isotherms at 77 K of KCMO.

The pore-size distribution, derived from the adsorption/desorption branches under equilibrium conditions, is shown in the Supporting Information (Figure S3) and depicts pores predominantly distributed in the mesoporous range. These textural features associated with the particle aggregation (SEM images) can influence electrolyte diffusion and ion transport during electrochemical processes. As discussed later, the charge-storage behavior of KCMO was evaluated using electrochemical measurements.

### 3.4 Vibrational Property

The factor group analysis of KCu_2_OH­(MoO_4_)_2_(H_2_O) was performed considering its crystal structure in the trigonal system with space group $C_{3i}^{2}$ and Z = 3 at room temperature. The number of expected normal vibrational modes was determined using the site symmetry method.^36^ Accordingly, the atoms are located at the Wyckoff sites listed in Table 3. The site symmetries of the H atoms were analyzed based on reference.^18^ Each molecule contains 18 atoms, resulting in a total of 54 atoms and 162 degrees of freedom.

**Table 3:** Site Symmetries of the Atoms and Vibration Modes in the $C_{3i}^{2}$ -Space Group^a^

Atom	Wyckoff sites	Site symmetry	Irreducible representations
Mo1	6c	*C*_3_	A_g_ + A_u_ + E_g_ + E_u_
Cu1	9d	*C*_i_	3A_u_ + 3E_u_
K1	3a	*S*_6_	A_u_ + E_u_
O1	18f	*C*_1_	3A_g_ + 3A_u_ + 3E_g_ + 3E_u_
O2	6c	*C*_3_	A_g_ + A_u_ + E_g_ + E_u_
O3	6c	*C*_3_	A_g_ + A_u_ + E_g_ + E_u_
H1	18f	*C*_1_	3A_g_ + 3A_u_ + 3E_g_ + 3E_u_
H2	6c	*C*_3_	A_g_ + A_u_ + E_g_ + E_u_

a Observation: The factor group analysis is based on the primitive unit cell. In this case, the number of formulas per unit cell is reduced by a factor of 3, *i.e.*, Z_B_ = Z/3 = 3/3 = 1.

The total number of freedom degrees is distributed in terms of the irreducible representation (considering the *C*_3*i*_ factor group) as Γ_Total_ = 10A_g_ + 10E_g_ + 14A_u_ + 14E_u_. Among these modes, two are acoustic Γ_Acoustic_ = A_u_ + E_u_. Therefore, the optically IR- and Raman-active modes are described by Γ_IR_ = 13A_u_ + 13E_u_ e Γ_Raman_ = 10A_g_ + 10E_g_, respectively.

Figure 4 shows the FT-IR spectrum of KCMO powder at 300 K, in the 400–3900 cm^–1^ range, highlighting the main bands and their respective assignments. The spectrum is in good agreement with those found in the literature for LTMs based on MoO_4_. The broad bands observed between 2900 and 3530 cm^–1^ are attributed to the symmetric (ν_s_(OH)) and asymmetric (ν_as_(OH)) stretching vibrations of hydroxyl groups.^21^ Bending modes of OH groups arise at ≈ 1630 cm^–1^.^19^ The characteristic IR bands associated with Mo–O bonds and hydroxyl groups coordinated to Cu are detected in the 1150–400 cm^–1^ interval.

**Figure 4:**
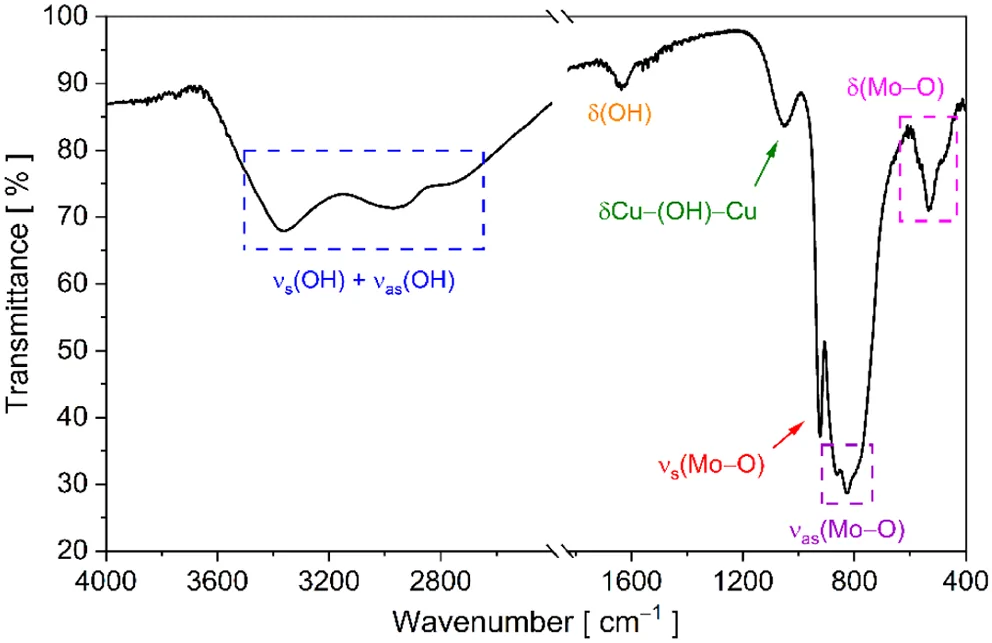
FT-IR spectrum of KCMO powder.

The band at ≈ 1052 cm^–1^ is attributed to the deformation of Cu–(OH)–Cu bonds,^37^ while the band at ≈ 923 cm^–1^ is associated with the ν_s_(Mo–O) symmetric stretching mode.^37^ The ν_as_(Mo–O) antisymmetric stretching modes of molybdate groups are found between 765 and 880 cm^–1^.^21,37^ Bands around 590–450 cm^–1^ are described as Mo–O deformation.^38,39^

The Raman spectrum of powdered KCMO, recorded in the 50–1100 cm^–1^ range at room temperature, is exhibited in Figure 5. Between 950 and 880 cm^–1^, modes associated with the symmetric stretching of Mo–O bonds are observed, while the asymmetric stretching appears in the 875–700 cm^–1^ range. Deformation modes of terminal Mo–O bonds are located between 560 and 190 cm^–1^. Signals below 170 cm^–1^ are described as lattice modes, also known as external modes, which are attributed to motions involving the entire crystal.^37^

**Figure 5:**
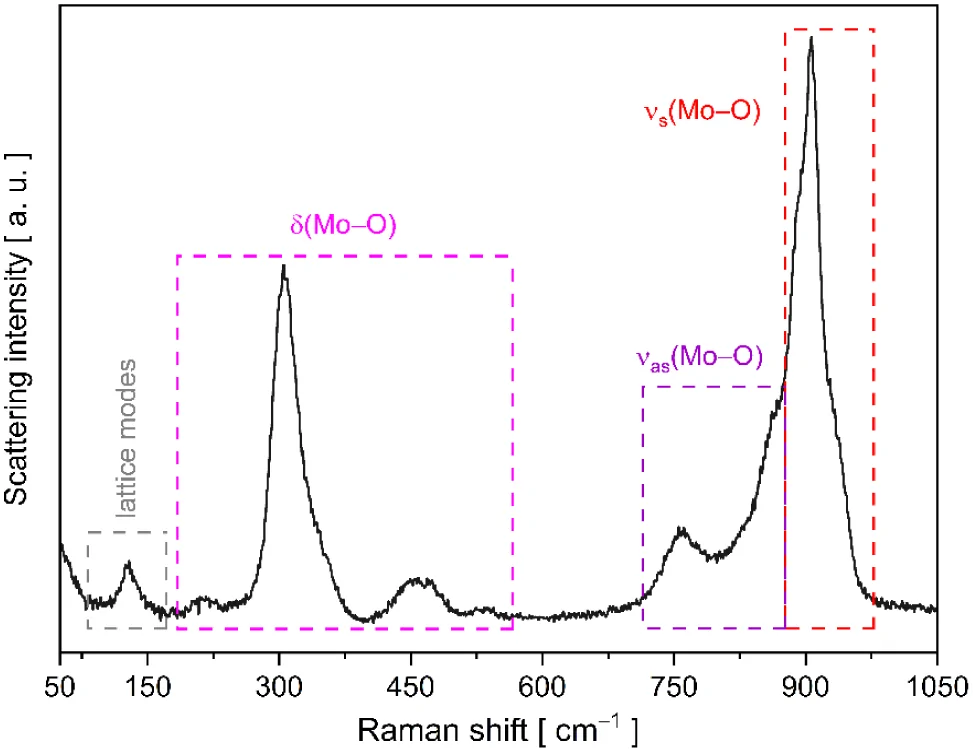
Raman spectrum of KCMO in the wavenumber region between 50 and 1100 cm^–1^.

### 3.5 Thermal Property

Figure 6 displays the TGA thermogram of KCMO. Mass loss occurs between 300 and 655 K, totaling ≈ 6.4% (≈ 33.3 g/mol), corresponding to the release of coordinated H_2_O molecules and structural dehydroxylation. This experimental value agrees with the theoretical mass calculated for the loss of one H_2_O molecule and one OH group from the KCMO unit. Between 655 and 850 K, the curve exhibits a thermal plateau, reflecting the formation of a stable intermediate phase. Beyond this temperature, a gradual additional mass loss suggests structural decomposition of the molybdate framework.

**Figure 6:**
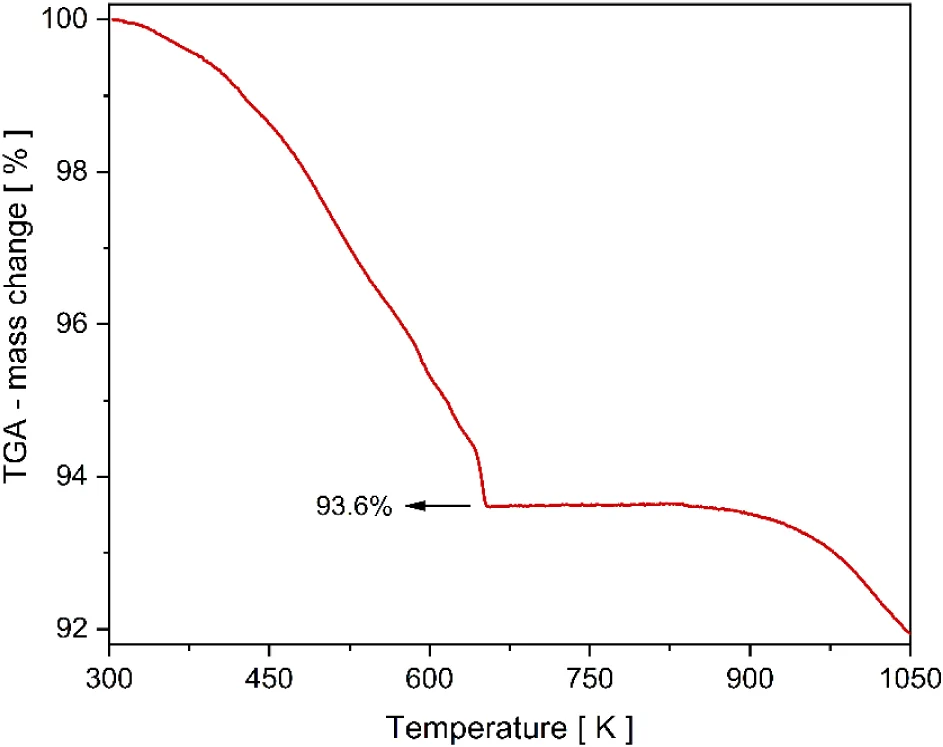
TGA thermogram of KCMO.

### 3.6 Electrochemical Studies

Figure 7a illustrates cyclic voltammograms of KCMO recorded at scan rates ranging from 5 to 50 mV/s, within the 0.0–0.4 V potential window using 3 M KOH electrolyte. The voltammogram profiles exhibit a well-defined pair of redox peaks at all scan rates, indicating reversible faradaic reactions and a charge-storage mechanism involving pseudocapacitive/battery-type behavior.^40,41^ The redox features are likely due to the electroactive Cu centers in the KCMO electrode, as commonly reported for copper-based pseudocapacitive materials.^29^ Moreover, as the scan rate increases, a shift in the redox peaks is observed, signaling an electrochemical reversibility of the KCMO electrode.^42^

**Figure 7:**
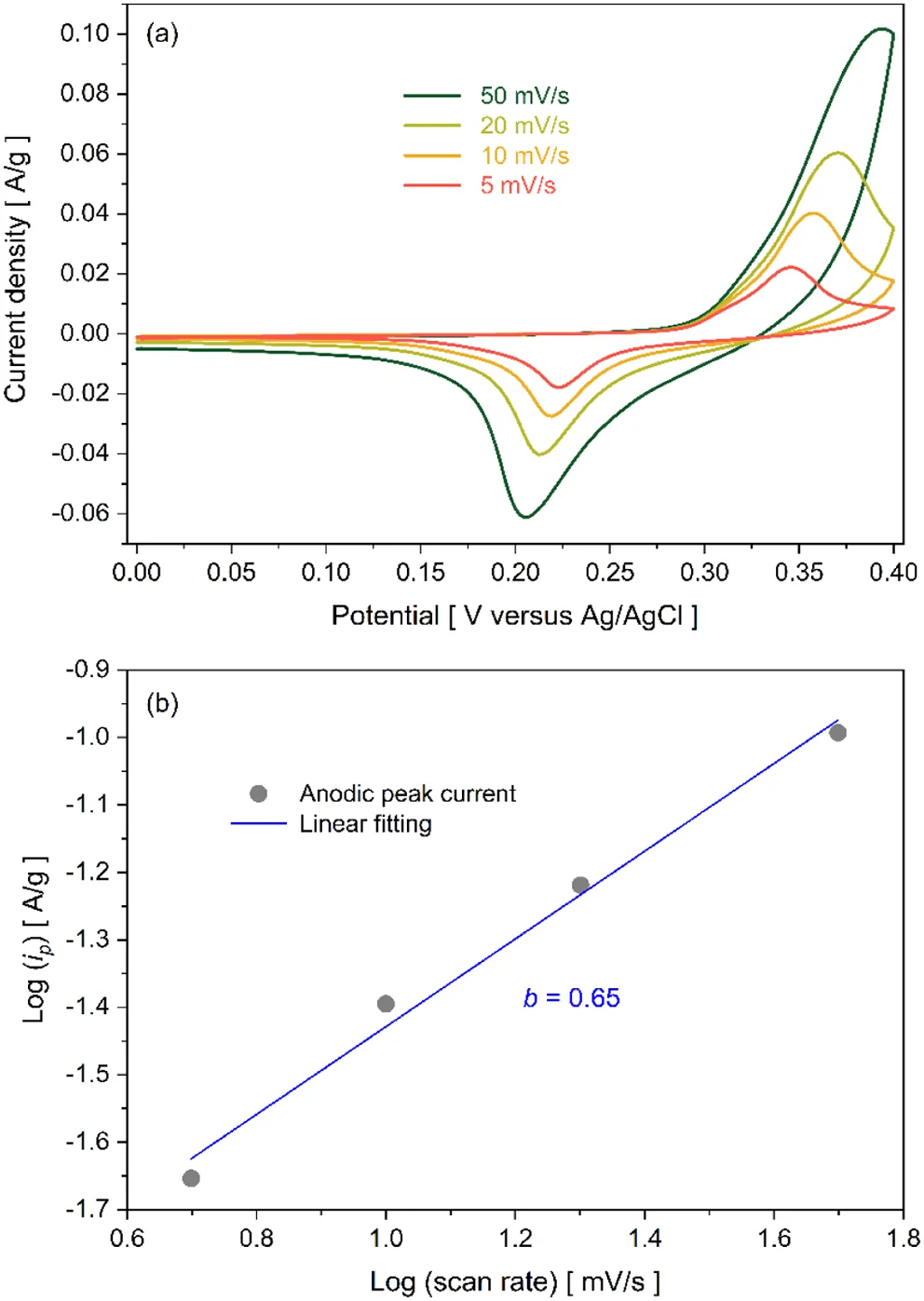
(**a**) CV curves and (**b**) Log (*i_p_*) versus Log (scan rate) plot of KCMO.

To further investigate the charge-storage kinetics, the correlation between the anodic peak current (*i*_*p*_) and scan rate (ν) was analyzed according to the *i*_*p*_ = *a*ν^*b*^ power-law. In this model, *b*-values ≈ 0.5 indicate diffusion-controlled faradaic behavior, whereas values close to 1.0 correspond to surface-controlled capacitive processes.^35^ The estimated 0.65 *b*-value (Figure 7b) suggests that the charge-storage mechanism involves diffusion-controlled with some pseudocapacitive contribution. Such a result is consistent with the redox behavior observed in the CV curves, characterizing mixed electrochemical kinetics for the KCMO electrode.

It is worth noting that textural features can influence the mixed electrochemical kinetics. In the KCMO case, the relatively low specific surface area and the presence of agglomerate particles, as evidenced by the BET and SEM analyses, can limit electrolyte accessibility and ion diffusion through the electrode material. The electrochemical response is therefore dependent on textural properties.

Figure 8a shows GCD curves obtained at current densities ranging from 1 to 10 A/g. The nonlinear charge/discharge profiles indicate faradaic redox reactions associated with pseudocapacitive/battery-type behavior.^43,44^ consistent with the CV redox features and the mixed electrochemical kinetics from the *b*-value analysis. Additionally, at higher current densities, the KCMO electrode exhibits faster charge/discharge processes, whereas at lower current densities, a better-defined charge/discharge profile is observed.

**Figure 8:**
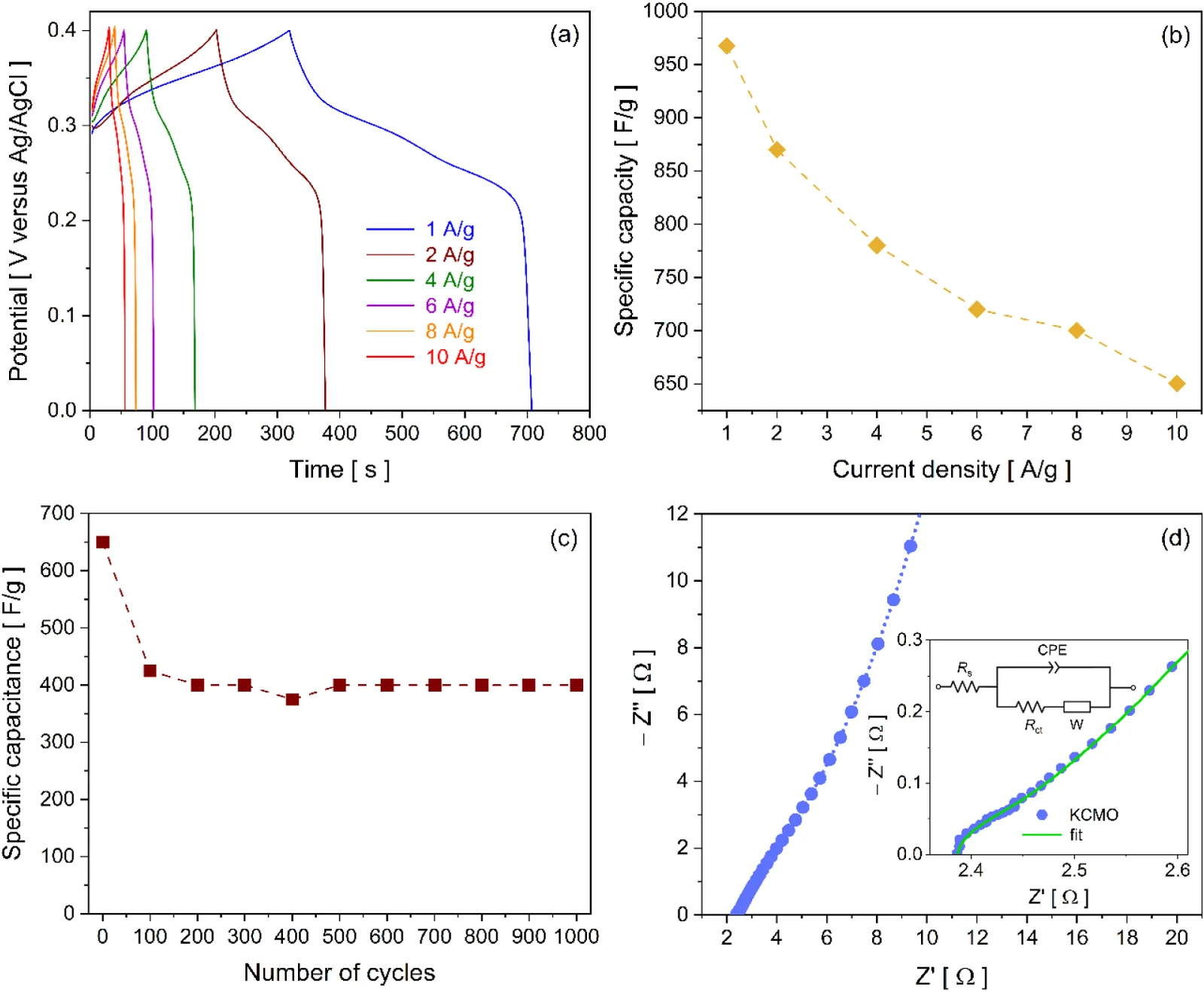
(**a**) GCD curves. (**b**) Specific capacitance as a function of current density. (**c**) Specific capacitance as a function of the number of cycles. (**d**) Electrochemical impedance spectrum. The inset figure illustrates a impedance fit in the low-real-impedance region, Z′. The equivalent circuit is also shown.

From the charge/discharge curves, the specific capacitance (*C*s) of the KCMO electrode was estimated at current densities ranging from 1 to 10 A/g (Figure 8b). The values are shown in Table 4.

**Table 4:** Specific Capacitance of the KCMO Electrode at Different Current Densities

	Specific capacitance	
Current density[A/g]	[F/g]	[mAh/g]
1	967	107
2	870	97
4	780	87
6	720	80
8	700	78
10	650	72

At a current density of 1 A/g, the electrode delivers a specific capacitance of 967 F/g (107 mAh/g). This performance is superior to that reported in the related literature, as summarized in Table 5, underscoring its potential as an electrode material for electrochemical energy storage systems. The cycling stability test showed that the KCMO electrode retained ≈ 62% of its initial specific capacitance after 1000 charge/discharge cycles at a current density of 10 A/g (Figure 8c). Although this retention indicates moderate cycling stability, the electrode remained electrochemically active throughout the test, suggesting that Faradaic and pseudocapacitive processes continued to contribute to charge storage. Postcycling SEM/EDS analyses (Figure S4, Supporting Information) showed that Mo, Cu, K, and O were still distributed over the electrode surface after 1000 cycles, confirming the persistence of the active material on the current collector. However, the postcycling XRD pattern (Figure S5, Supporting Information) was dominated by the intense diffraction peaks of the Ni foam current collector, and the characteristic reflections of pristine KCMO were not observed. Therefore, the conservation of the original crystalline phase could not be verified. This limitation is likely related to the small amount of active material deposited on the current collector, which prevents an unequivocal assessment of the crystallographic evolution of KCMO during electrochemical cycling. Consequently, the observed capacitance fading may be associated with structural degradation and/or morphological and interfacial changes induced by repeated redox processes; however, a direct correlation cannot be established from the present experimental evidence. Further improvements in cycling stability could be achieved through targeted electrode engineering strategies (symmetric/asymmetric supercapacitor devices), including surface modification, incorporation into conductive composites, and architectural optimization.

**Table 5:** Specific Capacitance Comparison Between the KCMO Electrode Reported Materials in the Literature

Material	Electrolyte	Specific capacitance [F/g]	Reference
Sn(MoO_4_)_2_	KOH	109	^45^
Bi_2_Mo_2_O_9_	KOH	197	^46^
γ-CuMoO_4_	LiOH	281	^29^
Cu_3_Mo_2_O_9_	KOH	283.9	^47^
MnMoO_4_	Na_2_SO_4_	424	^48^
FeMoO_4_	KOH	493	^41^
NiMoO_4_ hydrate	KOH	652	^49^
CoMoO_4_/GO	KOH	226	^50^
Cu_3_Mo_2_O_9_/MWCNT	KOH	553	^51^
MnMoO_4_/MWCNT	KOH	571	^28^
KCMO	KOH	967	This work

Electrochemical impedance data were acquired using Nyquist and Bode plots to investigate charge-transfer kinetics at the electrolyte/KCMO electrode interface. The Nyquist plot (Figure 8d) shows a quasi-semicircle in the high-frequency region, corresponding to a charge-transfer resistance, followed by a Warburg impedance (W) in the low-frequency region associated with ionic diffusion kinetics. The equivalent circuit (inset of Figure 8d) was selected for its physical relevance to pseudocapacitive electrodes and provides a reliable description of the charge-transfer resistance and nonideal capacitive behavior. A good agreement between the experimental and fitted impedance data is observed. The *R*_s_ value of 2.38 Ω corresponds to the solution resistance, which accounts for the ionic conductivity of the electrolyte and resistances within the experimental setup. On the other hand, the low *R*_ct_ value of 0.85 Ω indicates rapid charge-transfer kinetics and reflects resistance to faradaic reactions at the electrode/electrolyte interface. These parameters, (*C*_s_, W) for charge-storage capability and (*R*_s_, *R*_ct_) for power performance, are crucial for optimizing a KCMO-based electrode.^52^

## 4 Conclusion

The novel KCu_2_OH­(MoO_4_)_2_(H_2_O) compound was successfully synthesized via the sonochemical method. Its crystal structure was identified by PXRD and Rietveld refinement as a trigonal LTM polymorph belonging to the *R*3̅ space group. Furthermore, this material represents the first potassium-based analog to contain a monovalent cation other than NH_4_^+^, and it crystallizes in the ϕ_*y*_ phase. SEM analysis revealed a platelet-like microstructure. Electrochemical characterization suggested a mixed pseudocapacitive/Faradaic charge-storage mechanism, with the KCMO electrode delivering a specific capacitance of 967 F/g at a current density of 1 A/g, and stable performance (moderate) over prolonged charge/discharge cycling. The findings demonstrate that the material deserves further investigation. Strategies involving conductive composite engineering and electrode fabrication are expected to further enhance the electrochemical performance and practical viability of KCMO.

## Supplementary Material



## Notes

Comparison of reported layered transition metal molybdates (Table S1), photograph of the KCMO powder (Figure S1), particle size distribution (Figure S2), pore size distribution (Figure S3), postcycling SEM/EDS characterization of the KCMO electrode (Figure S4), postcycling X-ray diffraction patterns of the KCMO electrode in comparison with pristine KCMO, carbon black, and PVDF (Figure S5) (PDF)
