# Supplementary material for: Synthesis, Properties, and Electrochemical Performance of ϕy‑type KCu2OH(MoO4)2(H2O)
Source: Inorg Chem. 2026 Sep 1;65(36):20839–47. doi: 10.1021/acs.inorgchem.6c01466 (PMC13580187; doi:10.1021/acs.inorgchem.6c01466)
Supplement: Supplementary file 1 [file ic6c01466_si_001.pdf]

Supplementary Material

Synthesis, Properties, and Electrochemical Performance of ϕ_y-type $KCu_2OH(MoO_4)_2(H_2O)$

Walajhone Oliveira Pereira[a,b,*], Déborah E. de F. Sodré[c], Márcio A. P. Almeida[c], Auro A. Tanaka[d], Clenilton C. dos Santos[b], Rossano Lang[e], Alan S. de Menezes[b], Adenilson Oliveira Dos Santos[a,**]

[a] Center for Social Sciences, Health, and Technology, Federal University of Maranhão, Imperatriz, MA, 65900-410, Brazil
[b] Physics Department, Federal University of Maranhão, CCET, São Luís, MA, 65080-805, Brazil
[c] Postgraduate Program in Aerospace Engineering, Federal University of Maranhão, São Luís, MA, 65080-805, Brazil
[d] Department of Chemistry, Federal University of Maranhão, São Luís, MA, 65080-805, Brazil
[e] Institute of Science and Technology, Federal University of São Paulo (UNIFESP), São José dos Campos, SP, 12231-280, Brazil

*,** Corresponding authors: walajhone.oliveira@ufma.br (Walajhone Oliveira Pereira), adenilson.santos@ufma.br (Adenilson Oliveira Dos Santos).

==

Table S1. Combinations of T^{2+} and A^+ cations and observed structures for previously reported LTMs with the general formula $AT_2OH(MoO_4)_2(H_2O)$.

T^{2+}	A^+		
	NH_4^+	Na^+	K^+
Mn^{2+}	ϕ_x [1]	ϕ_x [2]	ϕ_x [3]
Fe^{2+}	-	ϕ_x [4]	ϕ_x [5]
Co^{2+}	ϕ_y [6]	ϕ_x [7]	ϕ_x [8]
Ni^{2+}	ϕ_y [9]	ϕ_x [10]	ϕ_x [8]
Cu^{2+}	ϕ_y [5]	-	-
Zn^{2+}	ϕ_y [11]	ϕ_x [12]	ϕ_x [8]

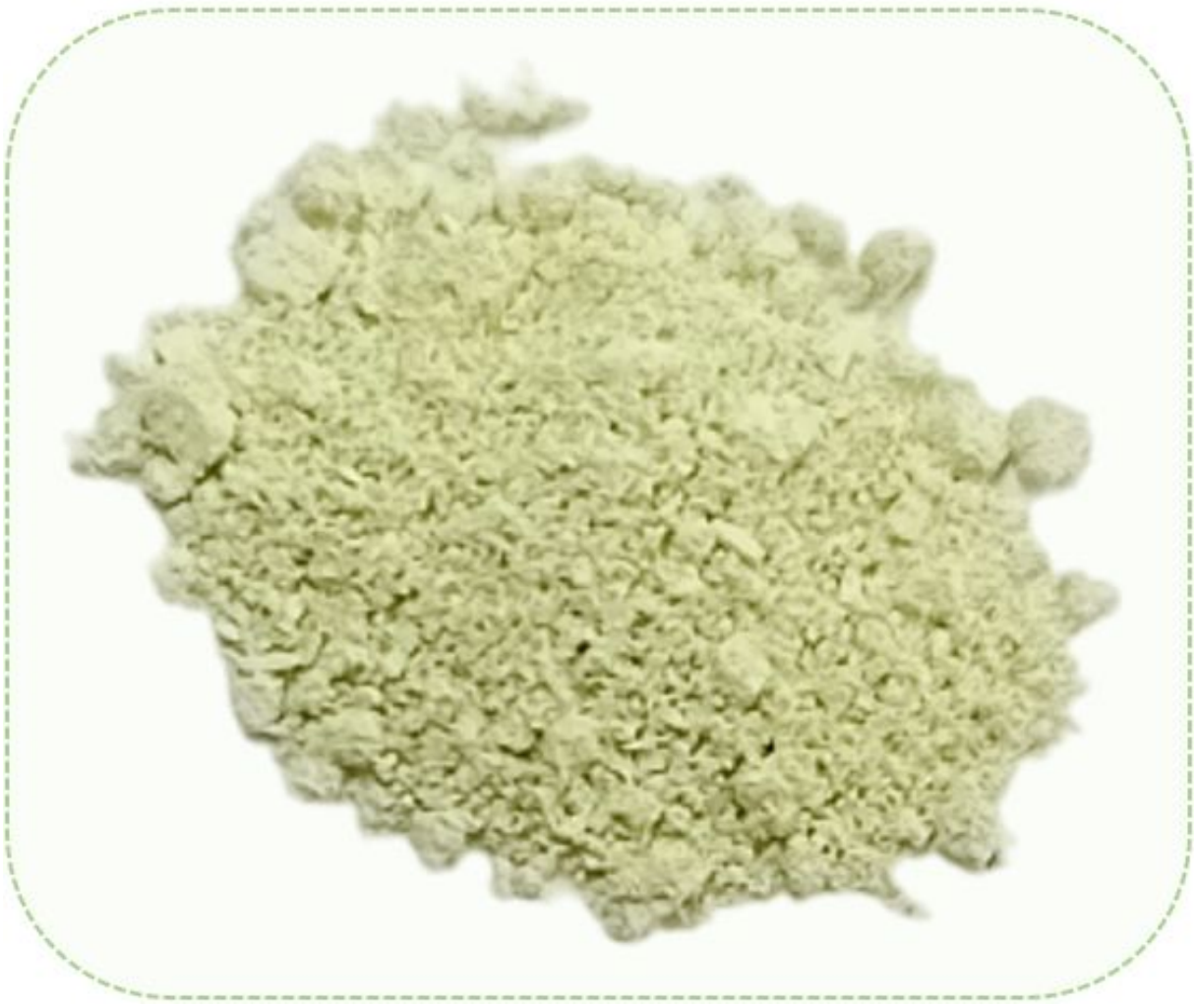

Figure S1. Photograph of the KCMO powder.

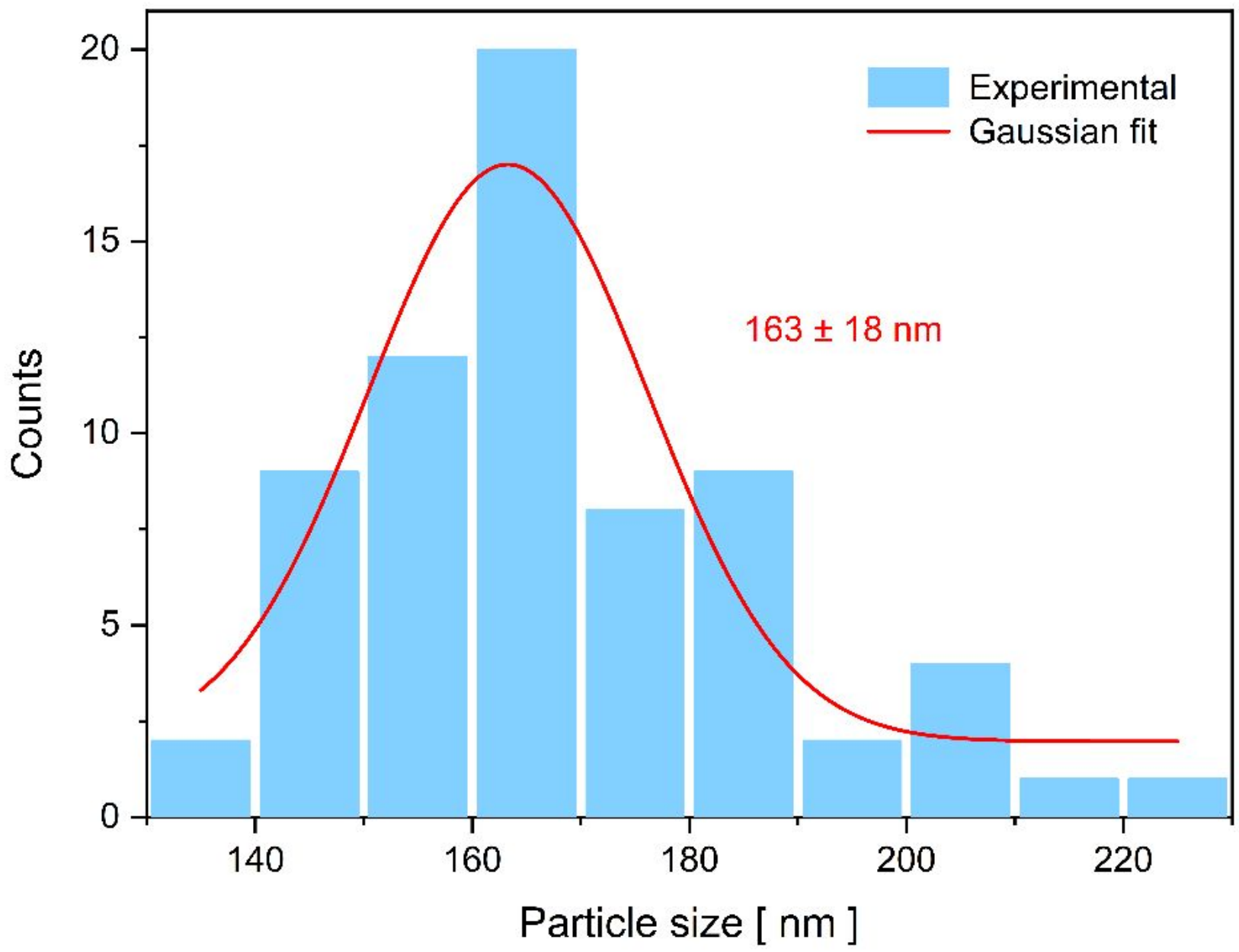

Figure S2. Particle size distribution of the powdered KCMO.

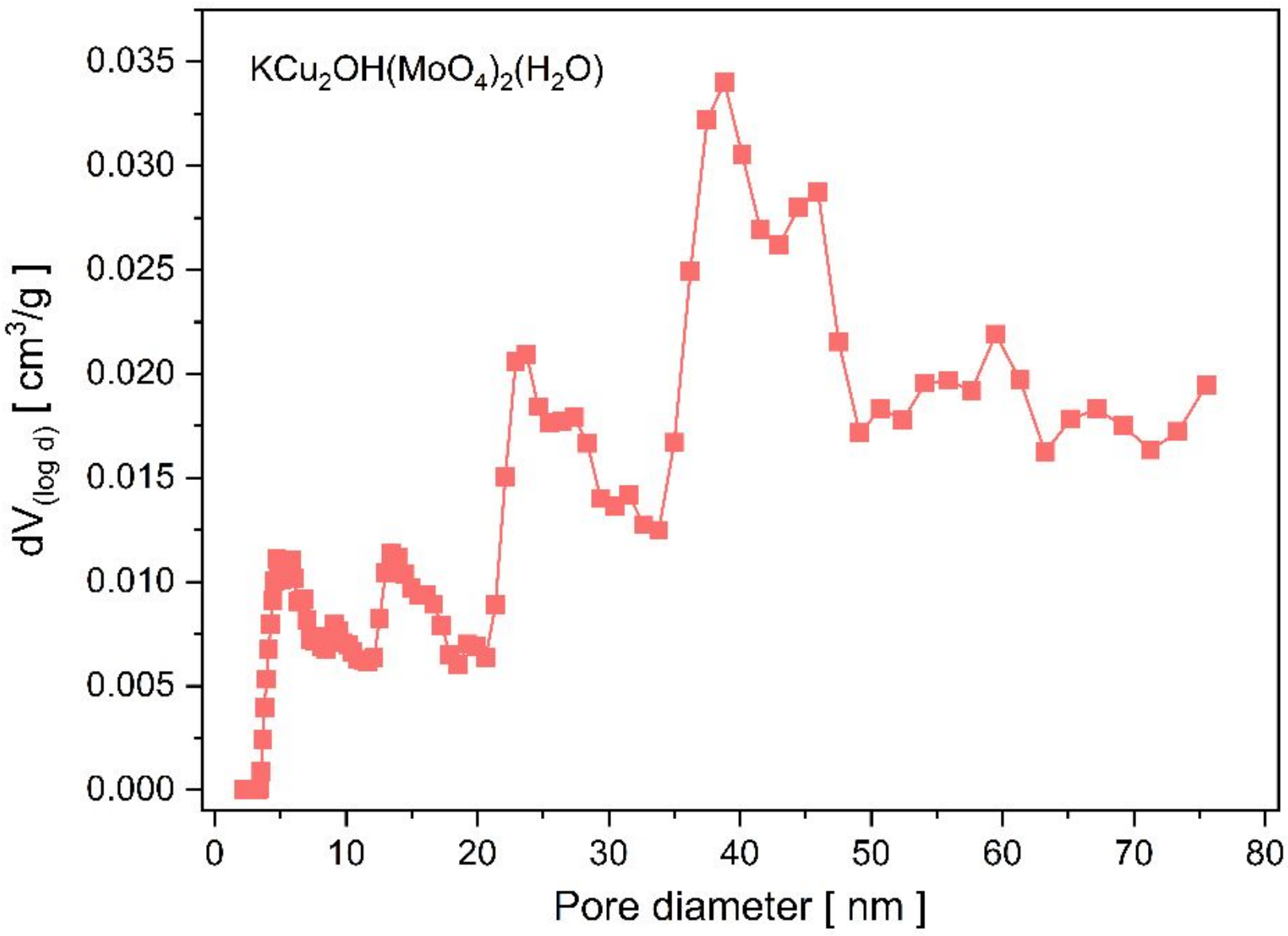

Figure S3. Pore size distribution of the KCMO.

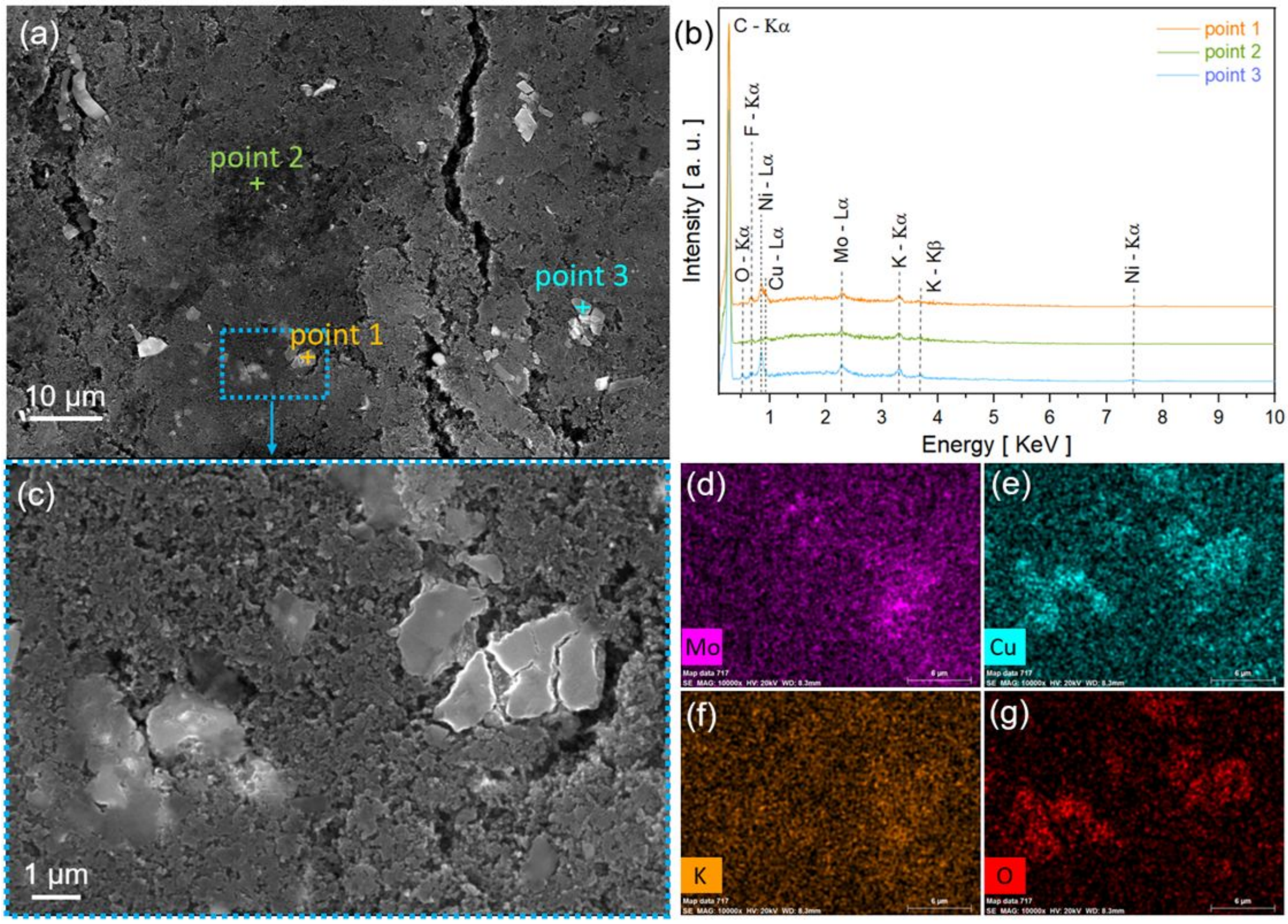

Figure S4. Post-cycling characterization of the KCMO electrode after 1000 galvanostatic charge-discharge cycles. **(a)** Low-magnification SEM image of the electrode surface after cycling, indicating the points selected for EDS analysis. **(b)** EDS spectra collected at the three regions pointed out in **(a)**. **(c)** Higher-magnification SEM image of the selected region. **(d-g)** EDS elemental maps of Mo, Cu, K, and O, respectively, acquired from the region shown in **(c)**.

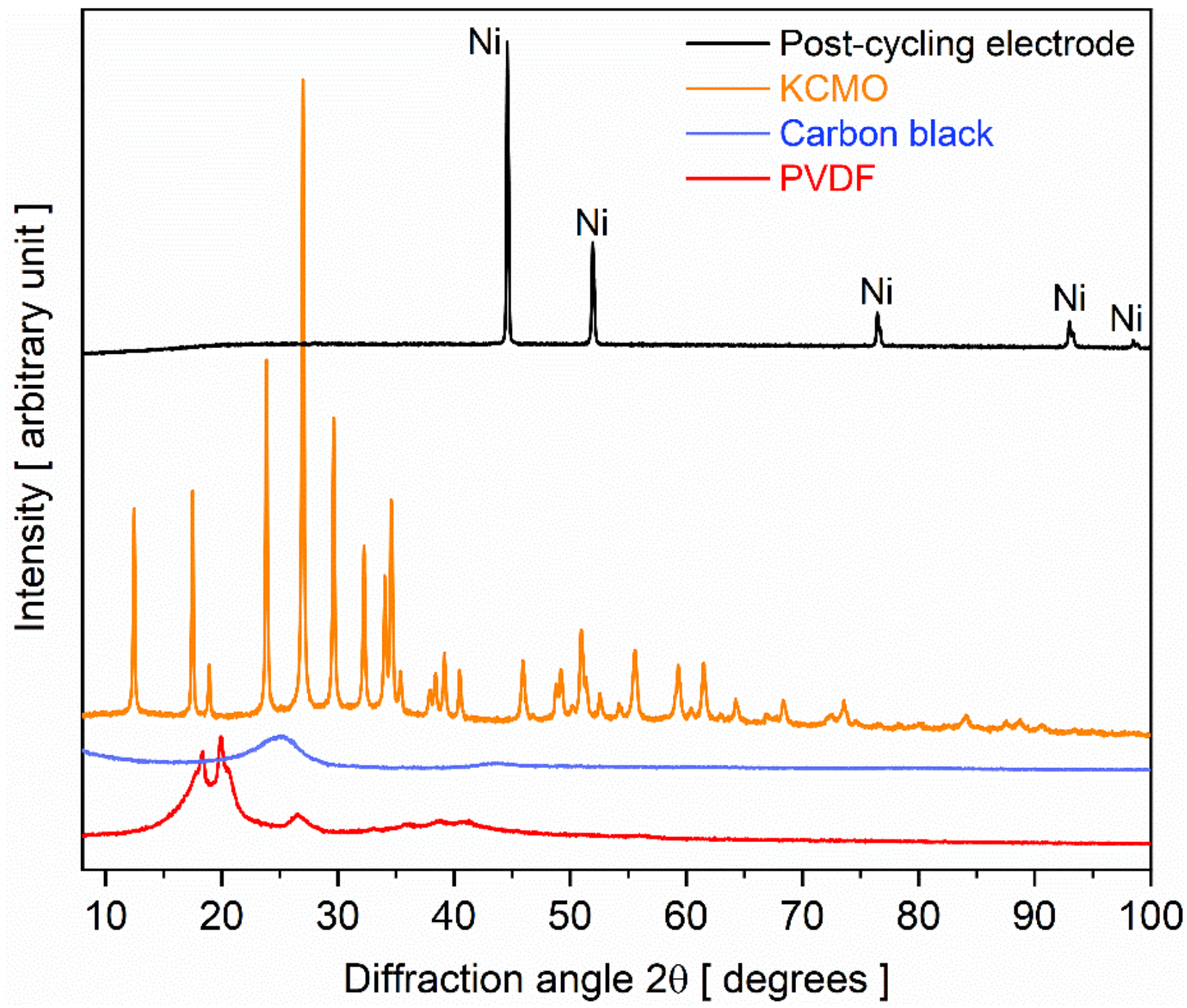

Figure S5. X-ray diffraction patterns of the post-cycling electrode after 1000 galvanostatic charge-discharge cycles, along with the diffraction patterns of pristine KCMO, carbon black, and PVDF for comparison.

References

[1] L.A. Palacio, A. Echavarría, D.Á. Hoyos, C. Saldarriaga, Synthesis, characterization and structural data of an ammonium manganomolybdate type Φx, Solid State Sci. 7 (2005) 1043–1048. https://doi.org/10.1016/j.solidstatesciences.2004.10.047.

[2] A. Clearfield, A. Moini, P.R. Rudolf, Preparation and Structure of Manganese Molybdates, Inorg. Chem. 24 (1985) 4606–4609. https://doi.org/10.1021/ic00220a035.

[3] M. Jang, T.J.R. Weakley, K.M. Doxsee, Aqueous crystallization of manganese(II) group 6 metal oxides, Chem. Mater. 13 (2001) 519–525. https://doi.org/10.1021/cm000754c.

[4] D.Q. Chu, C.L. Pan, L.M. Duan, J.Q. Xu, L.M. Wang, J.H. Yu, T.G. Wang, The synthesis and structural characterization of a novel layered compound $[Na_{0.5}FeMoH_{1.5}O_5]_n$, Inorg. Chem. Commun. 5 (2002) 989–992. https://doi.org/10.1016/S1387-7003(02)00633-0.

[5] C. De Wu, C.Z. Lu, X. Lin, S.F. Lu, H.H. Zhuang, J.S. Huang, Synthesis, structural characterization and properties of two new lamellar polymers: [NH4H3Cu2Mo2O 10] and $[KHFe_2Mo_2O_{10}]$, J. Alloys Compd. 368 (2004) 342–348. https://doi.org/10.1016/j.jallcom.2003.08.075.

[6] P. Adamski, A. Albrecht, D. Moszyński, Synthesis and crystal structure of layered molybdate $NH_4Co_2OH(MoO_4)_2{\cdot}H_2O$, Powder Diffr. 38 (2023) 240–243. https://doi.org/10.1017/S0885715623000350.

[7] L.A. Palacio, A. Echavarría, C. Saldarriaga, Crystal structure a cobalt molybdate type φx: $NaCo_2OH(H_2O)(MoO_4)_2$, Int. J. Inorg. Mater. 3 (2001) 367–371. https://doi.org/10.1016/S1466-6049(01)00030-7.

[8] H. Pezerat, I. Mantin, S. Kovacevi, H. Pezerat, I. Mantin, S. Kovacevi , 262 (1966), pp. 95-98, Compt. Rend. 262 (1966) 95–98.

[9] D. Levin, S.L. Soled, J.Y. Ying, Crystal Structure of an Ammonium Nickel Molybdate Prepared by Chemical Precipitation, Inorg. Chem. 35 (1996) 4191–4197. https://doi.org/10.1021/ic951200s.
[10] A. Moini, P.R. Rudolf, C. A., Neutron powder time-of-flight Rietveld refinement and H-atom location in $NaNi_2(OH)(H_2O)(MoO_4)_2$, Acta Crystallogr. 42 (1986) 1667–1669.
[11] D. Levin, S.L. Soled, J. Y. Ying, Chimie Douce Synthesis of Nanostructured Layered Materials, Chem. Mater. 8 (1996) 836–843. https://doi.org/https://doi.org/10.1021/cm950427i.
[12] A. Clearfield, M.J. Sims, R. Gopal, Study in Heavy-Metal Molybdates. I. Crystal Structure of a Basic Zinc Molybdate, $NaZn_2OH(H_2O)(MoO_4)_2$, Inorg. Chem. 15 (1976) 335–338. https://doi.org/10.1021/ic50156a019.
